# Sensing equivalent kinematics enables robot-assisted mirror rehabilitation training via a broaden learning system

**DOI:** 10.3389/fbioe.2024.1484265

**Published:** 2024-11-15

**Authors:** Qing Miao, Xueming Fu, Yi-Feng Chen

**Affiliations:** ^1^ School of Electrical and Electronic Engineering, Wuhan Polytechnic University, Wuhan, Hubei, China; ^2^ School of Biomedical Engineering, Division of Life Sciences and Medicine, University of Science and Technology of China, Hefei, Anhui, China; ^3^ Center for Medical Imaging, Robotics, Analytic Computing and Learning (MIRACLE), Suzhou Institute for Advance Research, University of Science and Technology of China, Suzhou Jiangsu, China; ^4^ Shenzhen Key Laboratory of Smart Healthcare Engineering, Guangdong Provincial Key Laboratory of Advanced Biomaterials, Department of Biomedical Engineering, Southern University of Science and Technology, Shenzhen, China

**Keywords:** robotic mirror rehabilitation, physical human-robot interaction, equivalent kinematics, broaden learning system, robotic control

## Abstract

**Introduction:**

Robot-assisted mirror therapy has been widely developed to help remodeling of premotor cortex for patients suffering from motor disability of limbs. Nevertheless, it is difficult to achieve real-time adaptive control in robot-assisted mirror rehabilitation training, particularly for patients with varying levels of limb impairment.

**Methods:**

This paper proposes an equivalent kinematics control framework based on the Broaden Learning System model for active robotic mirror rehabilitation, where people’s bilateral upper limbs actively perform mirror movements to enhance the impaired limb’s participation. The framework accommodates a broaden learning model from sensing multi-kinematic features to adjust the robotic damping coefficient in assisting human participants to complete mirror-symmetry training. Besides, in order to adapt to inter-patients’ variability with different disability levels, a challenge-level modification interface is also fused for safer training. This model is verified by additional symmetry indicator such as position trajectory error and force.

**Results:**

Experimental results show that the weaker subjects can also maintain mirror movement with the stronger subjects under the help of this model and verify the performance of framework in mirror-symmetry effects and movement smoothness. This leads us to believe that the framework can safely and efficiently assist human participants in completing mirror-symmetry movement.

**Discussion:**

The framework has the potential to improve outcomes in smoother and safer mirror-symmetry training by sensing multi-kinematic features. Future studies are necessary to involve clinical trials with actual patients.

## 1 Introduction

Increasing number of patients are suffering from limb movement disorders due to hemiplegia paralysis, which prevents them from completing activities of daily living (ADLs). Professional expertise and knowledge from physical therapists are generally required in traditional rehabilitation, which is labor-consuming and inefficient in terms of cost-effectiveness (Menner et al., 2020; Xu et al., 2020). Robot-assisted rehabilitation therapy has demonstrated the unprecedented potential to overcome these challenges in the past few decades, including nonlinear control techniques of soft robots for higher accuracy and safety (Cao et al., 2024b; Xie et al., 2024), human-in-the-Loop adaptive control concept for better adaptation of feedforward force and environment impedance in interaction tasks (Li et al., 2023), assist-as-needed control method for increasing muscle activity (Cao et al., 2024a), and so on.

As a clinical intervention, bilateral rehabilitation has been commonly used with positive clinical efficacy (Kantak et al., 2017; Richardson et al., 2021). Mirror therapy is one of the most classic methods in bilateral rehabilitation, which has shown the advantages of promoting the recovery of motor function, compared with conventional unimanual therapy (Summers et al., 2007; Dohle et al., 2009; Thieme et al., 2018). Mirror-symmetric bimanual therapy has also proven to be able to improve motion accuracy, range of motion, and flexibility (Stevens and Stoykov, 2003; Luft et al., 2004; Cauraugh and Summers, 2005). Traditional mirror therapy is a treatment that requires patients to visually feel the movement or touch of the uninhibited limb through optical illusion, thereby promoting the recovery of the impaired limb function (Stevens and Ellen Phillips Stoykov, 2004; Grünert-Plüss et al., 2008; Rothgangel et al., 2011). Traditional mirror therapy is generally delivered by physical therapists with passive or auxiliary activities.

Robot-assisted mirror therapy has attracted much attention in the past few decades due to enhanced productivity (Beom et al., 2016; Shahbazi et al., 2016; Xu et al., 2020; Zhang et al., 2022; Zhang C. Q. et al., 2023; Zhong et al., 2023). In an earlier study, Peter S. Lum et al. proposed a bilateral mode that robots lead patients bilateral upper limbs to perform mirror symmetry movements (Lum et al., 2005). Unfortunately, the patients passively complete the mirror training and the therapist cannot participate in patients’ training. In order for the therapist to play a full role in the patients’ mirror training process, M. Shahbazi et al. proposed a therapist-in-the-loop robot-assisted mirror therapy (Shahbazi et al., 2014; Shahbazi et al., 2015). In their framework, the haptic feedback about the patient’s impaired limb movement is provided to the therapists, who are required to guide a proper trajectory according to their expertise. The effectiveness and safety of robot-assisted mirror therapy were improved in M. Shahbazi’s study. However, over-reliance on the participation of therapists increases the labor cost of rehabilitation training. To save labor and realize in-home independent training, J. Xu et al. proposed a multi-channel reinforcement learning framework for lower-limb robotic mirror therapy, in which muscle activation and patient emotion replace therapists to guarantee the safety of subjects (Xu et al., 2020). J. Xu et al. proved that robotic mirror-symmetry training could achieve satisfactory rehabilitation performance in clinical experiments, showing the effect of robotic mirror training. The ideas proposed by these studies play an important role in the development of bilateral mirror training. Mirror training control strategy has also developed.

The bilateral impedance control strategy for mirror training in M. Shahbazi has been verified (Sharifi et al., 2017), however, it is difficult to determine impedance coefficients and the adaptive laws in practice. Though J. Xu et al. proposed a novel controller based on reinforcement learning, which could be used to find the optimal parameters of the impedance model, reinforcement learning requires a lot of trial and error to converge to ideal results. In this article, a novel equivalent kinematics control strategy based on supervised learning is proposed. In principle, reinforcement learning can be directly used for impedance adjustment (Rizzolatti and Craighero, 2004; Xu et al., 2020). However, when the human high-dimensional kinematic performance is considered, supervised learning has the advantage of requiring less online data and less time (Menner et al., 2020). Inspired by performance-based ideas (Krebs et al., 2003; Chemuturi et al., 2013; Papaleo et al., 2013; Baur et al., 2016; Leconte and Ronsse, 2016a), this article adopts the periodical kinematic features within a short time sliding window (such as average speed et al.) as supervised model’s inputs, which reflects the patients’ performance.

Recently, a fast and efficient network architecture, broad learning system (BLS), has been developed (Chen and Liu, 2018; Gong et al., 2022). Contrary to the deep neural networks that require a time-consuming training section to struggle for accuracy, the features of less time cost and competitive accuracy make BLS have great application potential in control (Feng and Chen, 2020; Gong et al., 2022). Huang et al. proposed an impedance learning framework (Huang et al., 2019; Huang et al., 2021), which solved the problem of robot-environment interaction based on BLS and fuzzy system. Another significant advantage of BLS is incrementally updating the weights of the network when newly obtained data need to be considered. These advantages of the BLS enable the controller to learn in a humanlike manner. Huang et al. proposed a novel framework based on BLS, which is proved to be valid for motor generalizing learning and accumulating knowledge to accomplish new tasks under the dynamic environment (Huang et al., 2020). Hence, the BLS was chosen as the learning strategy to control the impedance of the robot to achieve mirror rehabilitation training.

This paper aims to address the difficulty in achieving real-time adaptive control in robot-assisted mirror rehabilitation training, particularly for patients with varying levels of limb impairment. A control framework is designed that can adaptively adjust the robotic damping coefficient in real-time, optimizing mirror-symmetry movements, while ensuring smooth and safe interactions. Further, a BLS is used to dynamically sense and respond to the participant’s kinematic performance, which provides personalized, efficient, and scalable rehabilitation training.

Our contributions include: (1) an active robot-assisted mirror therapy framework that using healthy limb guiding impaired limb based on robotic adaptive assistance is proposed to promote the kinematics similarity between upper limbs. (2) a BLS model with a fast and smooth damping coefficient adjustment strategy is proposed to enhance the fluidity and safety of movements during mirror-symmetry rehabilitation training. The residual of this article is organized as follows. Section 2 presents the system description, the proposed control pipeline design, and the experiments. Then, Section 3 shows evaluation metrics and results. The discussion and conclusion are provided at last.

## 2 Methods

### 2.1 System description

**FIGURE 1:**
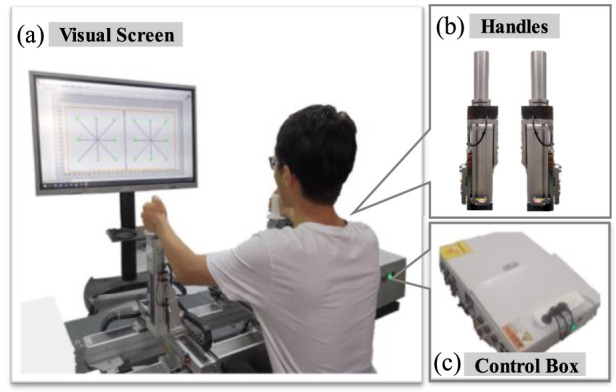
The bilateral upper limb training system. **(A)** the visual screen. **(B)** the robotic handles. **(C)** the control box of the robotic system.

The system is shown in Figure 1, which includes three degrees of freedom (DOFs) upper limb rehabilitation robot (only two degrees of freedom are adopted in this paper), and an extensive visual interface (Zhang M. M. et al., 2023). The robot mainly consists of three components, the three-axis force and position sensors, the control box and the two handles that can move in three-dimensional space. The real-time sensors’ position and force data can be sampled and recorded. Meanwhile, the real-time position will be displayed on the extensive visual interface in front of subjects to provide subjects with intuitive vision.

The robotic working mode is the human-active mode when the subject dominantly drives the robotic handles’ according to his subjective wishes and the adjustment of the robot handle’s damping coefficient can make both hands in different motion states. This work mode can significantly enhance the participation of subjects to improve motor function (Luo et al., 2019). The bilateral damping coefficients can be adjusted to change subjects’ upper limb movement difficulty.

The damping coefficient k (The healthy side’s $k^{H}$ decides the challenge level during training, and the impaired side’s damping coefficient $k^{I}$ will be adjusted to optimize the mirror-symmetry effects under a challenge level.) are generated according to our proposed supervised method. The max damping coefficient is limited by the maximum speed of the motor.

### 2.2 Framework method

The dynamic model of the robotic system interacted can be written by

$$M\ddot{q}+K\dot{q}=F_{h}$$ (1)

where $M=blockdiag\left\{M_{1},M_{2}\right\}\in R^{2n\times 2n}$ with $M_{i}=diag\;\left\{m_{i1},...,m_{in}\right\},i=1,2$ is the inertial matrix at end-effector, i=1 and i=2 separately denote left and right robotic handle, n=1 to n=3 respectively represent *X*-axis to *Z*-axis. $q={\left[q_{1}^{T},q_{2}^{T}\right]}^{T}\in R^{2n\times 1}$ with $q_{i}={\left[q_{i1}...,q_{in}\right]}^{T}\in R^{n\times 1}$ is the position of end-effector in Cartesian space. $F_{h}={\left[F_{h1}^{T},F_{h2}^{T}\right]}^{T}\in R^{2n\times 1}$ with $F_{hi}\in R^{n\times 1}$ is the interaction force applied on the end-effector by subjects, which can be measured by the force sensor.

In our robotic mirror rehabilitation, the damping coefficients of the handles can be adjusted to minimize the dissimilarity of bilateral kinematic performance. It is assumed that the subject’s unilateral limb movement ability is impaired (an impaired upper limb and a healthy limb). The optimization objective function can be defined as

$$L1=\sum_{i}^{n}\frac{1}{2}{\left(x_{i}^{I}-x_{i}^{H}\right)}^{2}$$ (2)

where the kinematic performance feature vector $X\in R^{n}$ , $x_{i}^{I}$ and $x_{i}^{H}$ refer to the $i^{th}$ feature of impaired upper limb performance $X^{I}$ , the $i^{th}$ feature of healthy upper limb performance $X^{H}$ .

The healthy limb, as the master, generates the appropriate kinematic performance $X^{H}$ under the fixed damping coefficient. The damping coefficient of the healthy limb can be modified to change the challenge level according to the training performance, which is friendly to supervisors. The fixed damping coefficient, selected according to the personalized conditions of subjects, determines the upper limit and bottom limit of the reference kinematics performance. A large damping coefficient makes it difficult for the impaired limb to keep up with the healthy limb’s movement, and a small damping coefficient maybe let subjects lose interest in training. When the impaired limb feels uncomfortable during training, the healthy limb can exert more muscle force to improve the performance to reduce the difficulty of the impaired limb under the framework.

**FIGURE 2:**
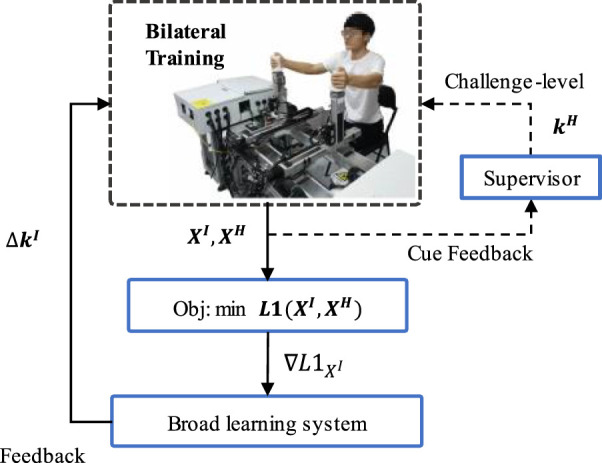
Overview of the control pipeline. The inner black solid line represents the data flow of the control pipeline; The outer orange dotted line indicates the challenge level modification interface that can adjustment training difficulty according to the participant’s different impaired level or mirror movement performance for personalized and safer rehabilitation training.

The impaired limb, as the slave, can keep up with the performance of the healthy limb with the assistance of the damping adjustment strategy based on proposed model. Figure 2 illustrates the proposed framework as a damping adjustment strategy. The input setting $k^{I}$ of the robot handle on the impaired side leads to the kinematic performance of impaired limb $X^{I}$ , as given in Equation 3.

$$X^{I}=g\left(k^{I}\right)$$ (3)

where $g\left(*\right)$ is an unknown function, the input setting $k^{I}$ are the damping coefficient of the impaired limb. The control target is to obtain the optimal input settings $k^{*}$ for which $X^{*}=g\left(k^{*}\right)$ is closest to the kinematic performance of healthy limb $X^{H}$ . In other words, the goal is to find a proper $k^{*}$ to minimize the objective function in Equation 1. In practical, we use incremental form: $∆X^{I}=g\left(∆k^{I}\right)$ .

The control strategy and conceptual ideas are as follows: the control pipeline is composed of the objective function reflecting the control objective, and the feedback section generates input adjustment $∆k^{I}.$ The objective function gives a scalar value reflecting the dissimilarity of kinematic performance of bimanual limbs. Therefore, the function provides a direction of improvement for the impaired feature vector, which is mapped to an input settings adjustment via the feedback section.

We define the control strategy for input adjustment $∆k^{I}$ as in Equation 4.

$$∆k^{I}=g^{-1}\left(∆X^{I}\right)-k^{I}$$ (4)

where $g^{-1}:R^{n}\to \;R^{1}$ is established by the broad learning system method (the inverse mapping of $g\left(*\right)$ in (3)). $∆k^{I}$ denotes the change of damping coefficient. In addition, $∆X^{I}$ refers to expected kinematic performance changes, denoted by the negative derivative of Equation 2: $-\nabla {L1}_{X^{I}}=X^{H}-X^{I}$ . In other words, the negative gradient of the target equation provides an improved direction for the feature vector $X^{I}$ . There the control law has the form in Equation 5.

$$∆k^{I}=f\left(X^{H}-X^{I}\right)-k^{I}$$ (5)

where $f:R^{n}\to \;R^{1}$ needs to be learned, play a role in making damping adjustments based on the state performance.

### 2.3 Broad learning system

The BLS, which is developed from a random vector functional-link neural network (Pao et al., 1994), provides a fast and efficient, and incremental learning way to learn and remodel in broad expansion without retraining the whole model.

In this learning system, the original input vectors are first mapped into random features through feature nodes by some feature mappings. Then, these features are sent into the “enhancement nodes” for nonlinear transformation to generate enhancement features. Finally, both the mapped features and enhancement features are fully connected to the output layer, and the full connection weights are the parameters that can be calculated rapidly by ridge regression of the pseudoinverse or can be optimized by gradient descent.

Given the training datasets $\left\{X,Y\right\}$ and n feature mappings $\phi_{i}$ , then the $i^{th}$ feature matrix can be described as in Equation 6.

$$Z_{i}=\phi_{i}\left(XW_{e_{i}}+\vartheta_{e_{i}}\right),i=1,2,\ldots ,n$$ (6)

where $X\in R^{N\times M}$ denotes the kinematic performance features (details are shown in Table 1 in Experiments Configuration Section). N is the number of input data, M is the number of features each sample, C is the dimension of outputs, weights $W_{e_{i}}$ and bias term $\vartheta_{e_{i}}$ are randomly initialized and need to learn. $\phi_{i}$ is the linear transformation function. In this work, the bias term is set to 0.

**TABLE 1:** Values for kinematic features vectors.

Symbol	Unit	Feature
$x^{1}$	mm/s	Mean velocity
$x^{2}$	mm/s	Maximum peak velocity
$x^{3}$	1	Number of peaks

The three kinematic indicators as the optimization objective of mirror symmetry movement, which show temporality and smoothness, are as follows (Cusmano et al., 2014): mean velocity, maximal peak velocity, and the number of peaks (only the velocity peaks bigger than 10% of the maximal peak velocity were considered).

We denote $Z^{n}≜\left[Z_{1},Z_{2},\ldots ,Z_{n}\right]$ as the sets of n groups of mapped feature nodes. The sparse autoencoder is utilized to generate the sparse features $Z^{n}$ of input. Then, $Z^{n}$ is fully connected to the enhancement nodes for nonlinear activation. Similarly, we obtain the outputs of the $j^{th}$ group of enhancement nodes by Equation 7.

$$H_{j}=\xi_{j}\left(Z^{n}W_{h_{j}}+\vartheta_{h_{j}}\right),i=1,2,\ldots ,m$$ (7)

where $\xi_{j}=\tanh\;\left(x\right)$ is the nonlinear activation function, weight $W_{h_{j}}$ and bias $\vartheta_{h_{j}}$ are randomly initialized. We denote the output matrix of the enhancement layer by $H^{m}≜\left[H_{1},H_{2},\ldots ,H_{m}\right]$ .

Therefore, the output of a BLS, the damping coefficient adjustment $\hat{Y}$ , can be denoted as in Equation 8.

$$\hat{Y}=\left(Z_{1},Z_{2},\ldots ,Z_{n},H_{1},H_{2},\ldots ,H_{m}\right)=W^{m}\left(Z^{n},H^{m}\right)W^{m}$$ (8)

where $W^{m}$ are the weights connecting the layer of mapped feature nodes and the layer of enhancement nodes to the output layer, and it can be easily obtained by the ridge regression approximation of pseudoinverse ${\left[Z^{n},H^{m}\right]}^{+}$ as in Equation 9.

$$W^{m}≜{\left[Z^{n},H^{m}\right]}^{+}Y$$ (9)

In the rehabilitation scenarios, the new training data is generated from the system after each training trial and we need to build a model that is easily adaptive to the new data or different subjects’ data. Retraining the model again with the whole training data is time-consuming. However, only by updating some weights can make the BLS be adapted to the new data, which is very practical.

Given that $\left\{X_{a},Y_{a}\right\}$ refers to the new training data for a BLS, where $X_{a}$ and $Y_{a}$ separately denote new input data and corresponding target output. The mapped feature nodes for $X_{a}$ can be written as in Equation 10.

$$Z_{a}^{n}=\left[\phi \left(XW_{e_{1}}+\vartheta_{e_{1}}\right),\ldots ,\phi \left(XW_{e_{n}}+\vartheta_{e_{n}}\right)\right]$$ (10)

where $Z_{a}^{n}$ denotes the feature nodes derived from the input data $X_{a}$ using a nonlinear mapping function ϕ . Then, the model’s output after processing the mapped feature nodes through the enhancement layer is denoted as in Equation 11.

$$A_{x}≜\left[Z_{a}^{n},\xi \left(Z_{x}^{n}W_{h_{1}}+\vartheta_{h_{1}}\right),\ldots ,\xi \left(Z_{x}^{n}W_{h_{m}}+\vartheta_{h_{m}}\right)\right]$$ (11)

where $Y_{a}^{T}$ denotes the true output, and $A_{x}^{T}W^{m}$ represents the predicted output. B is an adjustment factor. Final, the incremental weights are denoted as in Equations 12, 13.

$$W_{a}^{m}=W^{m}+\left(Y_{a}^{T}-A_{x}^{T}W^{m}\right)B$$ (12)

where

$$B^{T}=\left\{\begin{array}{c} C^{+},if\;C\neq 0\\ {\left(1+D^{T}D\right)}^{-1}{\left(A^{m}\right)}^{+}D,if\;C=0 \end{array}\right.\;C=A_{x}^{T}-D^{T}A^{m}\;D^{T}=A_{x}^{T}{\left(A^{m}\right)}^{+}$$ (13)

### 2.4 Control strategy based BLS

The modeling ideas and methods have been given before, and now we will begin to establish the control strategy. There are a series of data collection and processing tasks before training the model.

**FIGURE 3:**
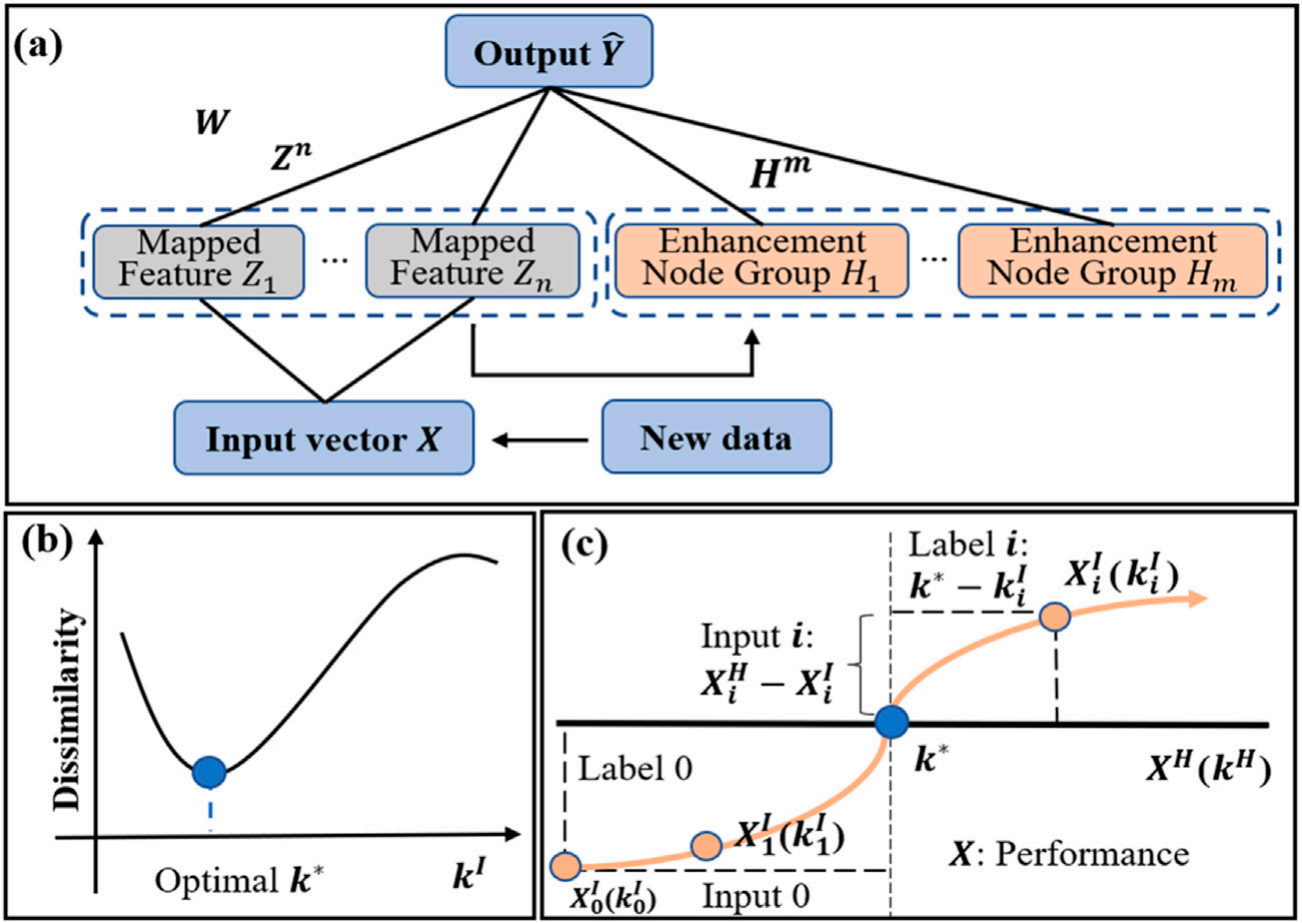
Top panel: **(A)** Structure of a BLS. Bottom panel: **(B)** Dissimilarity of kinematic performance between the impaired limb with the damping coefficient $k^{I}$ increasing from min to max and the healthy limb with the fixed damping coefficient. **(C)** How to convert the pretrain data into training data for BLS and label the data.

Generally speaking, there is a positive correlation between the damping coefficient and kinematic performance. Therefore, the dissimilarity of the kinematic performance of bilateral limbs has a trend as shown in Figure 3B with the impaired damping coefficient increasing from min to max, and there is an optimal $k^{*}$ (blue point) where the dissimilarity is minimum. The optimal $k^{*}$ converges to a certain value greater than the damping coefficient of the healthy side.

In the pre-train experiment, the kinematic performance of the impaired side changes with the increasing $k^{I}$ as shown in Figure 3C. The blue horizontal line represents the approximate kinematic performance of the healthy side under a fixed damping coefficient and the orange upward trend curve represents the kinematic performance of the impaired under the uniform increase of the damping coefficient $k^{I}$ . The intersection (blue point) of the two lines is the point with the smallest bilateral dissimilarity, which corresponds to the optimal $k^{*}$ . $X_{0}^{I}$ ( $k_{0}^{I}$ ) represents the performance $X_{0}^{I}$ under the damping coefficient $k_{0}^{I}$ . The input data is the performance difference $X_{i}^{H}-X_{i}^{I}$ and the label is $k^{*}-$ $k_{i}^{I}$ .

Algorithm 1. Train and Application of Feedback model.

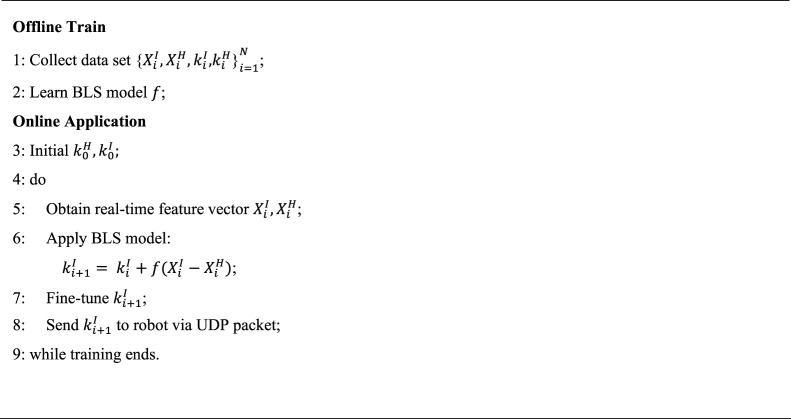

Remark 1: The selection of hyperparameters in the BLS model can use the grid search method, and at the same time, select a smaller number of neurons to prevent the model from overfitting when the accuracy is high.

Obtained the BLS model, we can achieve a whole closed loop control pipeline. We feed the input vector into the BLS model to get the damping adjustment, which is sent to the robot device in real-time via the UDP protocol after fine-tuning.

In actual training work, the damping adjustment predicted by the BLS model mainly considers how to quickly make the bilateral limbs achieve the same kinematic performance. Large damping adjustment will give rise to the unsmooth movement (when the damping adjustment exceeds the subject’s minimum sensitivity value, subjects will feel the sudden changes in damping coefficient and unsmooth movement). To solve the above deficiency, three tricks in the following, are integrated and applied to the output results of the BLS model to achieve fast and smooth damping adjustment.

Trick 1: Introduce a saturation function to limit the maximum value of each damping adjustment output $∆k^{I}$ from the BLS model. The function is as in Equation 14.

$$∆k=\left\{\begin{array}{l} ∆k,\left|∆k\right|<\;{∆k}_{limit}\\ +{∆k}_{limit},∆k\;>\;{+∆k}_{limit}\\ -{∆k}_{limit},∆k\;<\;{-∆k}_{limit} \end{array}\right.$$ (14)

where ${∆k}_{limit}$ is the limit of damping coefficient adjustment.

**FIGURE 4:**
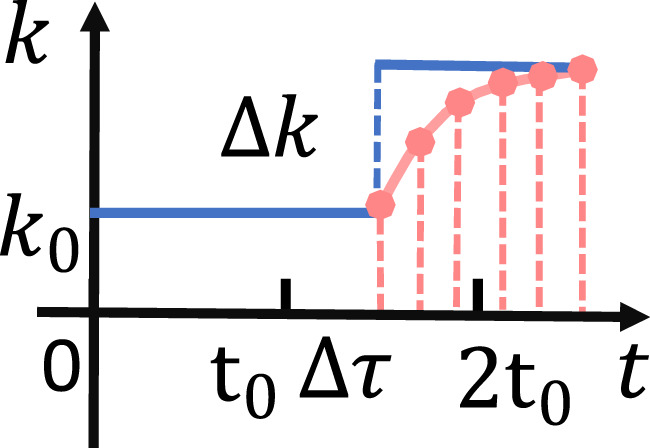
Interpolated and non-interpolated comparison. The blue solid line represents the sudden change of the damping coefficient in the case of non-interpolation. The pink point represents the slow change of the damping coefficient under the interpolation.

Trick 2: Perform n -time damping interpolation within the time sliding window T through logarithmic interpolation in Figure 4. The damping coefficients after interpolation are sent to the robot via the UDP package in turn. The interpolation calculation formula can be written as in Equations 15, 16.

$$stride=\frac{e^{1000*\left(k_{i}+∆k\right)}-e^{1000*k_{i}}}{num}$$ (15)

$$k_{i+j}=\ln\left(e^{k_{i}}+j*stride\right)\backslash 1000j\in \left(1,num\right)$$ (16)

where stride is interpolation’s step size, num is the number of interpolations. $k_{i}$ is the $i^{th}$ time window’s damping coefficient.

Trick 3: To increase the stability of the entire system, when the loss S is lower than the particular threshold, we reduce the damping adjustment by setting the decay factor $\delta \in \left(0,1\right)$ in Equation 17.

$$∆k^{I}=\left\{\begin{array}{l} ∆k,S\geq Threshold\;\\ \delta ∆k,S<Threshold\; \end{array}\right.$$ (17)

The fixed-step model is a method of fixing each adjustment’s step value without offline training compared with the supervised learning methods.

Fixed-step model: We also utilized the fixed-step model for comparison. There are only two options for adjusting the damping coefficient: increasing and decreasing $\left(+1,-1\right).$ We set the damping coefficient adjustment with a fixed step value, which is the maximum step value at which the subjects cannot feel the damping coefficient’s sudden change. The fixed-step control model focuses on the kinematic features to vote for deciding the sign of the damping coefficient. The formula can be written as in Equations 18, 19.

$$sign=\sum_{i=1}^{n}SIGN\left(x_{i}^{I}-x_{i}^{H}\right)$$ (18)

$$∆k=sign*∆k^{\prime }$$ (19)

where SIGN is a sign function; sign’s value is −1 or +1; $x_{i}^{I}$ and $x_{i}^{H}$ are the $i^{th}$ feature of the impaired and healthy upper limbs.

### 2.5 Experiments design

In this part, we show the design of the experiment for demonstrating the effectiveness of the method proposed in Part D. The experimental setup is composed of the bilateral robot device and two subjects with different upper limb strength imitating the impaired limb and the healthy limb of the patient with partial unilateral movement disorders. The user datagram protocol (UDP) was utilized to send data from the robot control box to control the program and send the damping coefficient from the control program to the robot control box. The communication frequency is 200 Hz.

#### 2.5.1 Experiment design

A total of nine healthy right-handed participants were enlisted in the experiment (height 155–178 cm, weight 43–66 kg, age 21-27). The study was approved by the Southern University of Science and Technology, Human Participants Ethics Committee (20,190,004), and consents were obtained from all participants.

**FIGURE 5:**
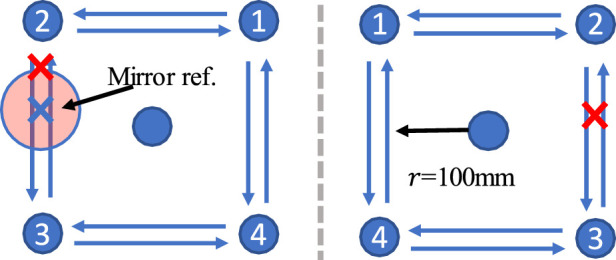
2x2 dot matrix visual trajectory. The radius of the square track is 100 mm. The impaired and health limb’s trajectories are symmetrical about the gray dotted line in the sagittal plane. The current position of the healthy side will be projected onto the affected side as a mirror trajectory reference.

Sitting straight in the chair, participants grip the two uncoupled handles with both hands and are instructed symmetrically to move the two handles along a square trajectory in the sagittal-transverse plane, respectively. In the experiment, we used a clockwise and counterclockwise square trajectory whose side length of 20cm, as shown in Figure 5. The participant’s real-time position information is displayed on a 65-inch large screen 1.5m away.

There are a total of three experiments, including a pre-train experiment to collect training data, a preliminary experiment to group subjects, and a formal experiment to verify the framework.

In the pre-train experiment to collecting training data ${\left\{X_{i}^{I},X_{i}^{H},k_{i}^{I},k_{i}^{H}\right\}}_{i=1}^{N}$ , the left handle’s damping coefficient is adjusted from the minimum value of 0.001 to the maximum value of 0.015 in the step of 0.0002 every 5s and the right handle’s damping coefficient is fixed. The subject was asked to apply steady force on the handles to move along the square trajectory spontaneously, and the movements of both hands did not interfere with each other. The collected training data can be used to train the BLS model after processing. The decay factor δ was set to 0.001, the limit of damping coefficient adjustment ${∆k}_{limit}$ was set to 0.003, and the threshold was set to 0.015.

The preliminary experiment was completed in advance to divide the subjects into two groups, the strong group, and the weak group. In the preliminary experiment, every subject was asked to complete a 1-min mirror movement along the square trajectory under the fixed damping coefficients which were different for males and females. The subjects were divided into two groups according to the average force level, the strong group and the weak group. We simulated the healthy upper limb on the right and the impaired upper limb on the left. The strong group’s subjects imitate the healthy limb and the weak group’s subjects imitate the impaired limb. In order to prevent two subjects from interfering with each other during training at the same time, the movement history of the strong group’s subject was recorded and reproduced in the formal experiment.

In the formal experiment, the subjects in the weak group need to put both hands on the two handles at the same time. However, he/she could only move the left handle, and the right handle moves spontaneously according to the recorded data. The task is to keep the mirror movement on both sides. In the case of inappropriate damping coefficients, subjects can perform a scaled-down version of the square trajectory movement according to their conditions.

When participants touch the handles, the handle’s kinematic data start to be recorded. In the beginning, if the left upper limb’s damping coefficient is difficult for the participant, the participant could choose to move in a small-scale trajectory.

#### 2.5.2 Experiments configuration

First, the raw data obtained by the pre-train experiment contains some abnormal data, noise data, and dirty data. Therefore, they need to be corrected before they can be used to train the BLS model. The processing includes correcting the training label, Box-cox transform, and data normalization. The offline data of two participants was used to train the BLS model, which was used for the formal experiments. The results show the good generalization of the supervised learning model.

In the formal experiment, each participant was instructed to complete nine formal experiments including three different reference trajectories as healthy side’s trajectories and three different assistant conditions (Unassisted, Fixed-step adjustment model, and BLS adjustment model). Each formal experiment lasts about 1 min, the order of the nine experiments is random, and there will be a 1-min rest after each trial. The initial left damping coefficient is the same as the damping coefficient in the preliminary experiment.

There are some details are shown as follows.

1) *Time Sliding Window:* the smallest sample unit. For real-time control, the kinematic features within a short time sliding window T are extracted. In our study, the time sliding window T is 1s, and it takes 0.05 s to extract data from UDP data stream to predict the adjustment $∆k^{I}$ on the computer with a 3.70 GHz Intel Core i7 CPU in Python (version 3.6) The total time of the time sliding window is strictly equal to the sum of the time to predict the new damping coefficient and the time to send the interpolated damping coefficients to the robot device. The prediction time includes data accumulation time, feature extraction time, and prediction time.
2) *Feature Vector:* the three kinematic features in Table 1 were computed from raw data and become the member of the participants’ kinematic performance feature vector. These kinematic variables were selected not only because they are usually used in clinical robotic studies but also relate to physiological aspects of the movement, which are easier to explain the effects of the training to patients.

## 3 Evaluation metrics and results

To evaluate the performance of the proposed framework, a previous clinical study was referred to define bilateral performance indicators (Colombo et al., 2014). The study recommended three kinds of robotic measured indicators to evaluate upper limb motor ability during reaching task, including mean velocity, movement accuracy and movement smoothness, as shown in Table 2. According to the results on 31 stroke patients and 15 healthy subjects, these indicators showed a very low error of measurements and coefficient of variation, which displayed positive clinical application values. The details are given as below.

1) Mean Velocity. The mean value of the velocity of the end-effector. In our study, this performance indicator was referred to define bilateral mean velocity error and bilateral maximum peak velocity error.
2) Movement Accuracy. This was assessed by measuring the mean absolute value of the distance of each point of the actual path travelled by the subject from the theoretical path. In our study, this performance indicator was referred to define bilateral force error and position error.
3) Movement Smoothness. Number of peaks in the tangential speed profile of a reaching movement, expressed as a negative value so that increases in the peak metrics equal increases in smoothness. In our study, this performance indicator was referred to define bilateral number of peak velocity error.

**TABLE 2:** Evaluation indicators of feedback model.

	Indicators
Optimization indicators for mirror evaluation	Bilateral mean velocity error
	Bilateral maximum peak velocity error
	Number of bilateral peak velocity error
Verification indicators for mirror evaluation	Bilateral force difference
	Bilateral trajectory position error
Indicators for smoothness evaluation	Smoothness of velocity
	SPARC

Note: SPARC, the spectral arc length is an accurate measure of movement smoothness for the movement speed. The indicators used in the evaluation of the mirror state are all bilateral indicator differences (Balasubramanian et al., 2012).

Two other indicators are selected to verify the smooth performance of the adjustment method in Section 2-D.

### 3.1 Evaluation of optimization indicators

**FIGURE 6:**
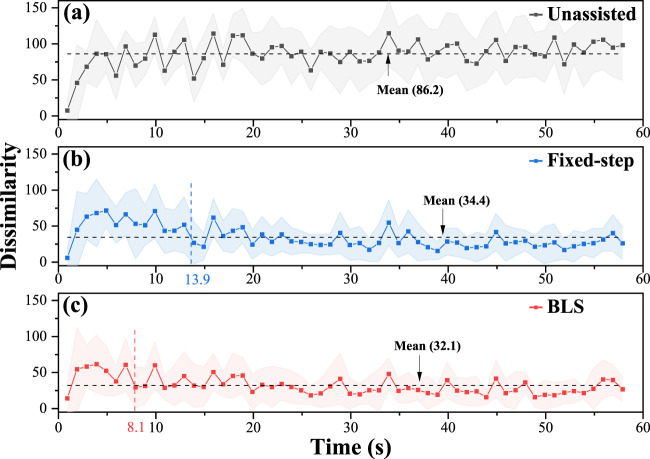
Evolution of the mean kinematics dissimilarity for six subjects under the three conditions in 60s. **(A)** the results of the unassisted situation. **(B)** the results of the fixed-step situation. **(C)** the results of the BLS.

**FIGURE 7:**
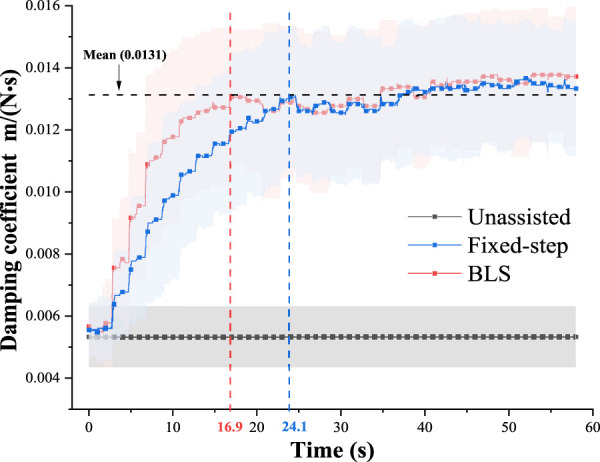
Evolution of the left damping coefficients ( $K^{I}$ ) in 60s.

The kinematics dissimilarity, which is the optimization objective of the proposed framework, reflects the subjects’ movement. Figure 6 shows the evolution of the mean kinematics dissimilarity for six subjects under the three conditions in the 60s. As can be seen, the dissimilarity of bilateral kinematics performance without assistance remains at a high value. Under the adjustment policy, the bilateral dissimilarity gradually decreases to a low stable value. The adjustment time delay is defined as the duration from the beginning of adjustment to the state that dissimilarity reaches around the mean value. The results are given in Figures 6, 7 show that the adjustment time delay of the BLS method is smaller than the Fix-step method.

From the adjustment delay perspective, the BLS method can rapidly assist participants in completing mirror-symmetry movement on average. When patients feel that the impaired limb is uncomfortable or painful, he/she can adjust the kinematic performance of healthy limb to help the impaired limb relieve the trouble instantaneously. (Such as reducing movement speed).

The evolution of the left damping coefficients ( $K^{I}$ ) in 60s under three different conditions are shown in Figure 6. The results show that the supervised learning modeling method based on the participant training data is effective.

**FIGURE 8:**
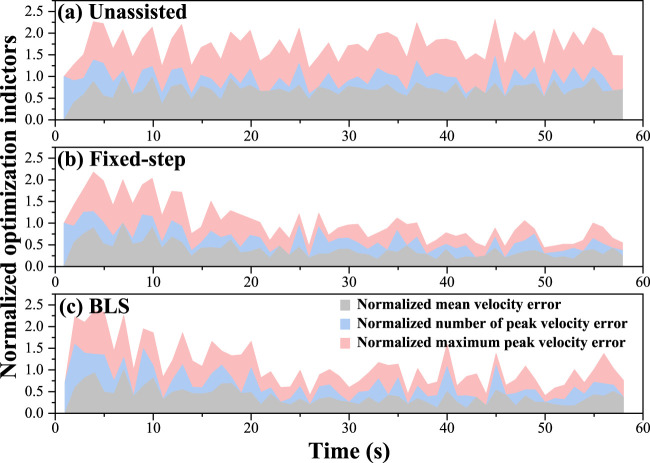
Evolution of mean velocity, number of peak velocity and maximum peak velocity in 60s. **(A)** the results of the unassisted situation. **(B)** the results of the fixed-step situation. **(C)** the results of the BLS.

Three optimization indicators, including mean velocity error number of peak velocity error, and maximum peak velocity error, are used to analyze the effect of kinematics equivalent mirror-symmetry movement. As is seen in Figure 8, the Fixed-step model and BLS model can significantly reduce the difference in bilateral upper limbs’ kinematic performance error, especially the mean velocity error and the maximum peak velocity error. The “number of peak velocity error” did not increase significantly, indicating that the adjustment did not cause the subject’s movement to be unsmooth.

According to the optimization and comprehensive indicators’ evaluation results, it can be inferred that the proposed framework can achieve a great mirror symmetry effect without causing unsmooth movement. Further verification results are given in Section 3-B.

Meanwhile, we analyze variance on the dissimilarity of six participants under three different conditions in Figure 8. The test results show that both the Fixed-step method and BLS method can significantly reduce the difference of bilateral upper limbs’ performance error.

### 3.2 Evaluation of verification indicators

**FIGURE 9:**
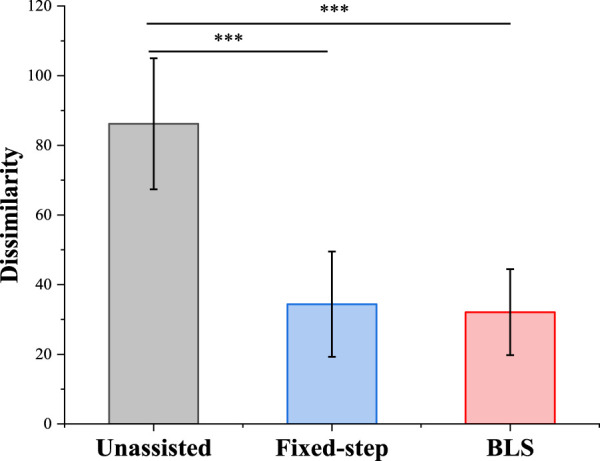
The analysis of variance of three method for mean Kinematics-Similarity. * indicates statistical significance: *p < 0.05, ** *p* < 0.01 and *** *p* < 0.001.

To verify the effect of the proposed framework, the mirror position error (mirror the real-time position of one side to the opposite side and calculate the Euclidean distance) and the difference of bilateral force are adopted. The error of bilateral position under three different conditions is shown in Figure 9. As can be seen, the position error has converged to low values under these adjustment models. It can be seen from the verification results that the Fixed-step method and BLS method can assist subjects with weak strength in achieving great mirror-symmetry effects.

**FIGURE 10:**
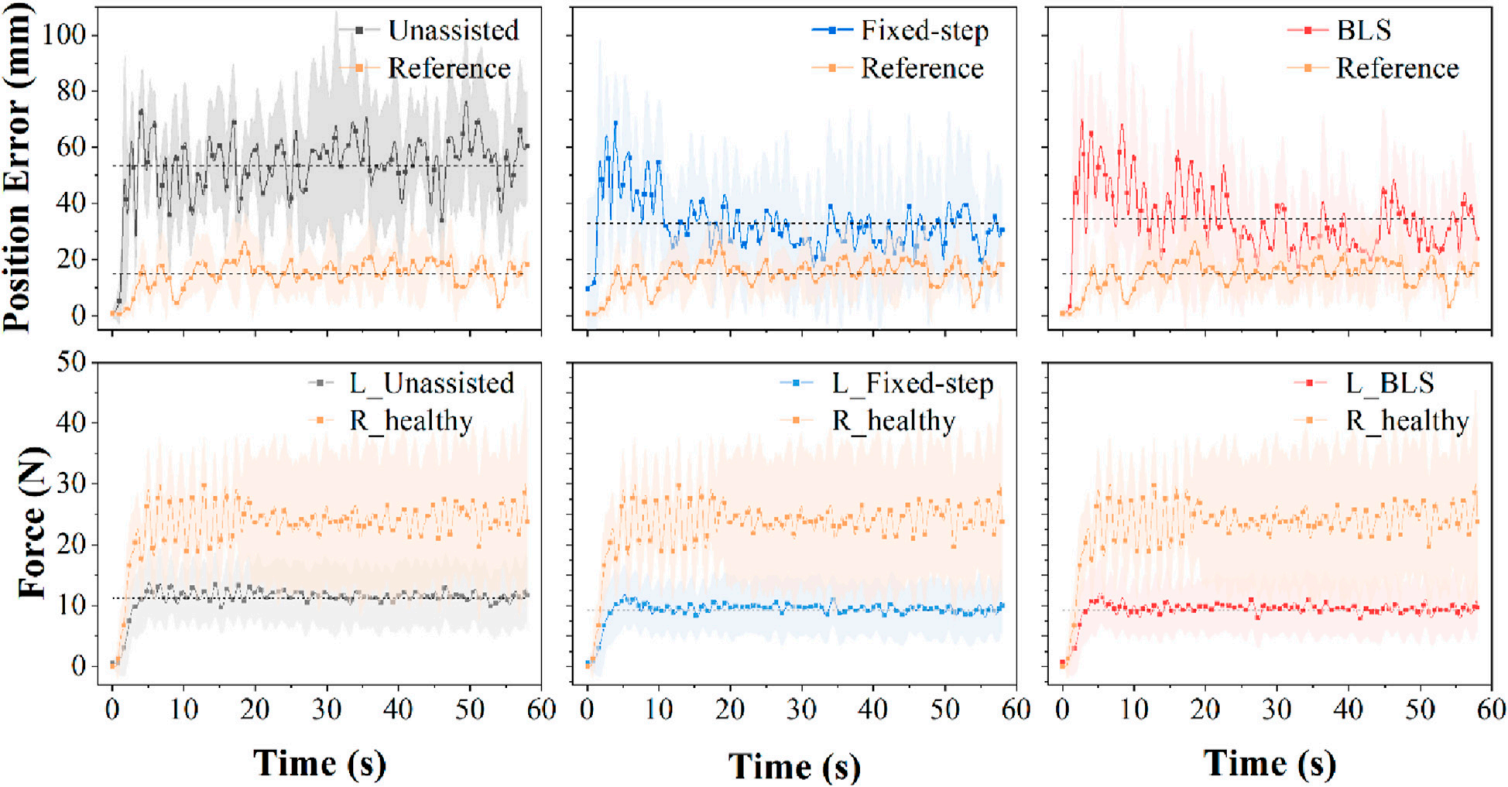
Top panel: Evolution of position error of six participants under three conditions in 60s. Bottom panel: Evolution of force of six participants under three conditions in 60s. *p* < 0.05.

The difference of bilateral force under three different conditions is shown in Figure 10. Under the three conditions, the average force levels of the weak subjects were similar. Combining the results of the two verification indicators in Figures 9, 10, the proposed method has the potential to help patients with unilateral motor dysfunction complete mirror rehabilitation training.

### 3.3 Evaluation of movement smoothness

**FIGURE 11:**
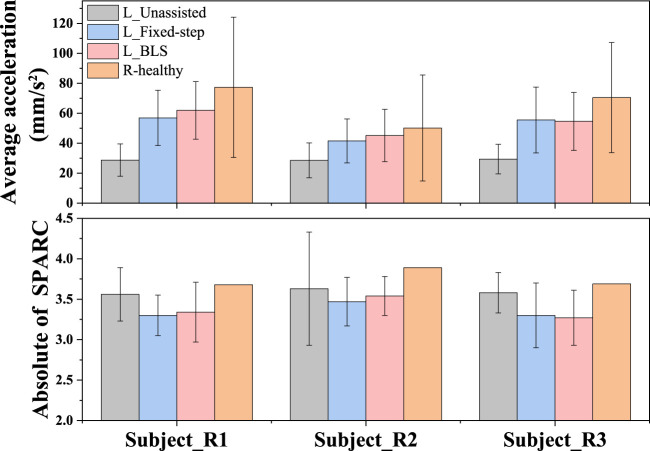
Comparison of the weak subjects’ movement smoothness under the three conditions and the reference subjects’ movement smoothness. The average acceleration indicates the volatility of speed. The Absolute of SPARC is the measure of the smoothness of movement speed in the frequency domain. The low Absolute of SPARC value indicates high smoothness of movement speed.

Patients with unilateral limb dyskinesia are accompanied by rhythmic and discrete movements (Montes et al., 2014; Leconte and Ronsse, 2016b). Thus, there is no doubt that movement smoothness is also an indispensable consideration to promote the recovery of a patient’s motor function (Krebs et al., 1998; Balasubramanian et al., 2012). There is a tradeoff between the damping coefficient adjustments and movement smoothness. To observe the influence of the damping coefficient’s adjustment on the smoothness of the participants’ bilateral upper limbs under the two different conditions, average acceleration, and the SPARC are compared in Figure 11. With the help of the damping adjustment strategy, the subjects’ average speed variability is closer to those of the strong group. The auxiliary strategy based on the BLS model can make the impaired side’s volatility of speed closer to that of the healthy side compared to the adjustment method based on the fixed-step model. From the SPARC indicator, the adjustment of damping can make the weaker subjects move more smoothly. As can be seen, the proposed framework allows the weaker subjects to move more naturally and smoothly like the stronger ones.

Although the proposed model has not been applied to clinical experiments, the experimental results show this model’s good performance in ordinary people’s simulation experiments. Our future work will focus on conducting clinical trials to evaluate the effectiveness of the proposed robotic mirror rehabilitation.

## 4 Discussion and conclusion

In this study, we proposed an active robot-assisted mirror rehabilitation framework that incorporates an equivalent kinematics control strategy, powered by a Broad Learning System (BLS). The framework enables bilateral mirror movements, facilitating the rehabilitation of patients with impaired upper limbs. By sensing multi-kinematic features, the system adjusts the robotic damping coefficients online, ensuring that patients experience smoother and safer mirror-symmetry training. The experimental results showed that the BLS model enabled fast adaptation and good synchronization between the healthy and impaired limbs during mirror-symmetry training. Key metrics such as mean velocity error and peak velocity error showed significant reductions, indicating that the system could better maintain bilateral symmetry. Furthermore, the dynamic adjustment of damping coefficients contributed to smooth movements, minimizing abrupt changes and enhancing the overall comfort and efficiency of the rehabilitation process.

The BLS model has advantages in two aspects compared with deep learning models as below.

- Fast Offline Training and Lower Complexity. Unlike deep neural networks (DNNs) that require extensive iterative training and high computational resources, BLS performs efficient offline training. It uses random feature mapping and enhancement nodes that do not require gradient-based backpropagation. Only the weights between the enhancement nodes and the output layer are learned using the Moore-Penrose pseudoinverse, which is computationally less intensive. BLS is a flat network, meaning that increasing its width (adding more nodes) grows the number of parameters far less than increasing depth in DNNs. As cited by (Gong et al., 2022), this flat structure allows for a more efficient scaling of the model with lower computational demands compared to deep networks, which are typically deeper and more computationally intensive. This makes BLS advantageous when real-time predictions need to be generated frequently.
- Incremental Learning Capability. A key feature of BLS is its incremental learning capability. This allows the model to update quickly with new data or enhancement nodes without needing to retrain the entire network. In real-time control scenarios, such as patient rehabilitation, this is particularly useful because the model can adapt incrementally as the patient’s condition changes. BLS’s adaptability makes it an ideal candidate for such applications, as it handles incremental data without needing to retrain the entire network, unlike deep learning architectures, which would increase computational complexity. For example, a previous study demonstrated that BLS was effective for edge computing applications like traffic analysis systems, where the model could adapt to new data without being fully retrained (Xiting et al., 2019). Similarly, in rehabilitation scenarios, BLS can be incrementally adjusted to reflect changes in a patient’s progress, offering flexible and adaptive control without requiring a complete retraining process.

Despite these promising results, there are some limitations that need to be addressed. On one hand, the current experiments were conducted with only healthy participants simulating motor disturbance rather than real patients. Nevertheless, the system can adjust the damping coefficient in real-time to optimize mirror symmetry and includes a challenge-level modification interface, which adapts to the varying abilities of different individuals. Additionally, the system allows the adjustment of the healthy limbs’ movements to reduce the difficulty for the impaired limb, which can help alleviate the burden during rehabilitation. Hence, clinical trials with actual patients are applicable to evaluate the full potential of this approach. On the other hand, the comparison between different advanced control methods will be involved in the future work as well.

## Data Availability

The original contributions presented in the study are included in the article/supplementary material, further inquiries can be directed to the corresponding authors.
